# Supplementary material for: Recovering directional brain networks under temporal undersampling: an application to schizophrenia
Source: bioRxiv. 2026 Aug 14:2026.08.09.743708. Preprint. [Version 1] doi: 10.64898/2026.08.09.743708 (PMC13640626; doi:10.64898/2026.08.09.743708)
Supplement: Supplementary file 1 [file NIHPP2026.08.09.743708v1-supplement-1.pdf]

bioRxiv preprint doi: https://doi.org/10.64898/2026.08.09.743708; this version posted August 14, 2026. The copyright holder for this preprint (which was not certified by peer review) is the author/funder, who has granted bioRxiv a license to display the preprint in perpetuity. It is made available under a CC-BY 4.0 International license.

7 Supporting Information

7.1 Clingo encoding and implementation of sRASL and RnR

This section connects the graph-theoretic definitions in Eqs. (1)–(2) to their computational implementation. The distinction between sRASL and RnR is important. Solver-based RASL (sRASL) encodes the noiseless inverse problem: it searches for causal-timescale graphs $\mathcal{G}^1$ and undersampling rates u for which the graph implied at the measured timescale is exactly the supplied graph $\mathcal{H}$ [1]. Real-world noisy RASL (RnR) retains the same forward undersampling rules, but replaces exact agreement with weighted optimization because a graph estimated from finite fMRI data need not be the exact undersampled image of any $\mathcal{G}^1$ [2]. Both methods are implemented in the open-source `gunfolds` package [35] using the Answer Set Programming solver `clingo`.

7.1.1 Why Answer Set Programming is appropriate

The recovery problem in Eq. (2) requires inversion of a many-to-one mapping. Given a causal-timescale graph $\mathcal{G}^1$ and an undersampling rate u, the forward operator in Eq. (1) determines the measured graph $\mathcal{G}^u$. In the reverse direction, however, a single measured graph can have multiple causal-timescale explanations. Recovery must therefore search over both the space of directed graphs and the possible undersampling rates.

For n nodes, there are n^2 possible directed edges when self-connections are allowed and consequently 2^{n^2} possible causal-timescale graphs. A procedural algorithm must generate candidates, calculate their undersampled graphs, compare each result with $\mathcal{H}$, and implement specialized pruning rules. Adding new structural information then requires modifying the search procedure itself.

Answer Set Programming (ASP) provides a natural alternative because it is declarative. Rather than specifying how to traverse the candidate space, the program specifies what constitutes a candidate and what conditions make it valid. The solver is responsible for finding the objects that satisfy those conditions. The forward undersampling operator, agreement with the observed graph, strongly connected component restrictions, the density prior, and the edge-evidence weights can therefore be expressed as separate, composable logical statements.

bioRxiv preprint doi: https://doi.org/10.64898/2026.08.09.743708; this version posted August 14, 2026. The copyright holder for this preprint (which was not certified by peer review) is the author/funder, who has granted bioRxiv a license to display the preprint in perpetuity. It is made available under a CC-BY 4.0 International license.

This representation is particularly appropriate here because the scientific answer is generally a set of graphs rather than a unique point estimate. In exact sRASL, the answer sets returned by the solver correspond to the members of the equivalence class in Eq. (2), within the represented rate domain and modelling assumptions. RnR uses the same representation but ranks feasible explanations according to their disagreement with a noisy estimated graph.

7.1.2 The ASP language used in the encoding

An ASP program is commonly organized according to a generate–define–test structure. Facts describe the particular problem instance. Choice rules generate candidate objects. Ordinary rules derive the consequences of each candidate. Integrity constraints eliminate candidates that violate the model. In RnR, weak constraints replace strict rejection with weighted penalties.

The principal ASP constructs used in the implementation are as follows:

- **Facts** are unconditional statements describing the problem instance. For example, the measured directed edge $1 \to 3$ can be represented by `hdirected(1,3).`, while `hbidirected(2,7).` represents $2 \leftrightarrow 7$ in $\mathcal{H}$. A fact such as `scc(1,2).` assigns node 1 to strongly connected component 2. The shorthand `node(1..n).` produces one fact for every node.
- **Ordinary rules** have the form `Head :- Body.`. The rule means that the head must hold whenever the body holds. Variables begin with uppercase letters and are instantiated over their finite domains during grounding. For example,

  ```
  directed(X,Y,1) :- edge1(X,Y).
  ```

  states that a candidate edge from X to Y is a directed walk of length one.
- **Choice rules** generate the candidate space. The rule

  ```
  {edge1(X,Y)} :- node(X), node(Y).
  ```

  allows each possible causal-timescale edge to be present or absent. Cardinality bounds can restrict the number of selected atoms. The rule

  ```
  1 {u(1..maxu)} 1.
  ```

  selects exactly one undersampling rate for each answer set.
- **Integrity constraints** have an empty head and take the form `:- Body.`. A candidate is rejected whenever the body is true. Exact sRASL uses integrity constraints to require the graph derived at the selected rate to equal $\mathcal{H}$.
- **Weak constraints** have the form

  ```
  :~ Body.  [W@P,...]
  ```

  and do not reject a candidate. Instead, a satisfied weak constraint adds weight W to the candidate's objective at priority P. `clingo` minimizes higher priority levels before lower ones. RnR uses weak constraints to penalize disagreements with noisy or uncertain features of $\mathcal{H}$.

The central graph predicates have direct interpretations:

- `edge1(X,Y)` denotes $X \to Y$ in the unknown causal-timescale graph $\mathcal{G}^1$.
- `u(U)` denotes the candidate undersampling rate.

bioRxiv preprint doi: https://doi.org/10.64898/2026.08.09.743708; this version posted August 14, 2026. The copyright holder for this preprint (which was not certified by peer review) is the author/funder, who has granted bioRxiv a license to display the preprint in perpetuity. It is made available under a CC-BY 4.0 International license.

- `directed(X,Y,L)` states that $\mathcal{G}^1$ contains a directed walk of length L from X to Y. At $L = u$, this implies $X \to Y$ in $\mathcal{G}^u$.
- `bidirected(X,Y,U)` states that X and Y have a common ancestor that reaches both through directed walks of the same length $L < U$. This implies $X \leftrightarrow Y$ in $\mathcal{G}^U$.
- Predicates beginning with `h`, such as `hdirected` and `hbidirected`, describe the measured input graph $\mathcal{H}$ rather than a candidate causal graph.

After grounding the variables over the finite node and rate domains, `clingo` searches the resulting propositional program for stable models, or "answer sets." In this application, the displayed atoms in one answer set encode one candidate graph–rate pair $(\mathcal{G}^1, u)$.

7.1.3 Generating a candidate causal graph

The following ASP fragment generates the space of candidate causal-timescale graphs and possible undersampling rates. The constant `maxu` is the maximum rate considered by the solver.

```
#const n = 10.
#const maxu = 20.

node(1..n).

1 { u(1..maxu) } 1.

{ edge1(X,Y) } :- node(X), node(Y).
```

The first choice rule selects exactly one rate. The second independently selects the presence or absence of every possible directed edge in $\mathcal{G}^1$. Consequently, the rule represents the full candidate space without requiring a manually written loop over graphs.

Self-connections are included because an edge $X^{t-1} \to X^t$ represents temporal persistence of the same variable and can affect both directed reachability and SCC stability.

7.1.4 Encoding the forward undersampling operator

The following rules derive the directed and bidirected edges that a candidate $\mathcal{G}^1$ would produce after undersampling. They constitute a direct discrete encoding of Eq. (1).

```
directed(X,Y,1) :-
    edge1(X,Y).

directed(X,Y,L) :-
    directed(X,Z,L-1),
    edge1(Z,Y),
    L <= U,
    u(U).

bidirected(X,Y,U) :-
    directed(Z,X,L),
    directed(Z,Y,L),
    node(X;Y;Z),
    X < Y,
    L < U,
    u(U).
```

bioRxiv preprint doi: https://doi.org/10.64898/2026.08.09.743708; this version posted August 14, 2026. The copyright holder for this preprint (which was not certified by peer review) is the author/funder, who has granted bioRxiv a license to display the preprint in perpetuity. It is made available under a CC-BY 4.0 International license.

The first rule establishes the base case: every candidate edge is a directed walk of length one. The second rule recursively extends directed walks. If X reaches Z in $L-1$ steps and $Z \to Y$ is an edge of $\mathcal{G}^1$, then X reaches Y in L steps. Therefore, `directed(X,Y,U)` holds exactly when the candidate graph induces $X \to Y$ at the selected rate U.

The final rule implements the hidden-common-cause component of undersampling. It derives a bidirected edge between X and Y when some node Z reaches both through directed walks of equal length $L < U$. The common cause lies between measured time points and is therefore unobserved at the measured timescale. The restriction $X < Y$ stores each unordered bidirected pair only once.

These rules are shared by exact sRASL and RnR. The difference between the methods lies primarily in how the derived graph is compared with $\mathcal{H}$.

7.1.5 Exact matching in sRASL

For an error-free measured graph, sRASL enforces equality between the derived graph $\mathcal{G}^u$ and $\mathcal{H}$. The following four integrity constraints exclude, respectively, an extra directed edge, an extra bidirected edge, a missing directed edge, and a missing bidirected edge:

```
:- directed(X,Y,U),
   not hdirected(X,Y),
   u(U).

:- bidirected(X,Y,U),
   not hbidirected(X,Y),
   X < Y,
   u(U).

:- not directed(X,Y,U),
   hdirected(X,Y),
   u(U).

:- not bidirected(X,Y,U),
   hbidirected(X,Y),
   X < Y,
   u(U).
```

The first two constraints prevent the candidate from producing edges that are absent from $\mathcal{H}$. The final two prevent the candidate from omitting edges that are present in $\mathcal{H}$. Together they impose

$$\mathcal{G}^u = \mathcal{H}.$$

Every answer set that survives these constraints is therefore a valid pre-image of $\mathcal{H}$ under the undersampling operator, within the finite rate domain and the assumed model class.

This exactness is also a limitation when $\mathcal{H}$ is estimated from finite data. Statistical noise can add, omit, or misorient edges, and only a subset of mixed directed–bidirected graphs can be generated by undersampling a purely directed $\mathcal{G}^1$. An estimated graph may consequently have no exact pre-image, in which case the exact ASP program is unsatisfiable.

7.1.6 Strongly connected component constraints

sRASL improves computational efficiency by encoding structural information about strongly connected components (SCCs). Facts of the form `scc(X,K)` assign node X to component K, while facts such as `dag(K,L)` record permitted directions between components. A representative SCC restriction is

```
:- edge1(X,Y),
   scc(X,K),
   scc(Y,L),
```

bioRxiv preprint doi: https://doi.org/10.64898/2026.08.09.743708; this version posted August 14, 2026. The copyright holder for this preprint (which was not certified by peer review) is the author/funder, who has granted bioRxiv a license to display the preprint in perpetuity. It is made available under a CC-BY 4.0 International license.

```
    K != L,
    sccsize(L,S),
    S > 1,
    not dag(K,L).
```

Cycles remain permitted within an SCC, but connections between different SCCs must be compatible with the component-level directed acyclic graph. Under the loop-length condition stated in the Methods, SCC membership is stable under undersampling. Supplying the SCC facts before search therefore eliminates large families of structurally incompatible candidates.

These restrictions are one reason sRASL is substantially faster than search-based RASL. Instead of generating a graph and discovering only after undersampling that its component structure is impossible, the ASP solver incorporates the structural restrictions during search. The present analysis uses the domain-constrained SCC encoding described in Section 3.1.

7.1.7 The finite rate domain

The executable program searches over a finite range of rates controlled by `maxu`. This is both a modelling decision and a computational necessity. Repeatedly undersampling a finite graph does not produce new graphs indefinitely. Under the same loop-length condition that stabilizes SCC membership, the sequence eventually reaches a repeated or stable state [36].

The implementation can therefore suppress later occurrences after the compressed graph repeats an earlier state. Exact enumeration is complete relative to the represented rate range and the stated structural assumptions. The upper bound should be selected using domain knowledge about the relationship between the measurement interval and the plausible causal timescale. It is not itself learned continuously from the data; rather, the solver selects a discrete rate within the specified range.

7.1.8 RnR as a weighted relaxation of sRASL

RnR retains the graph generator and forward undersampling rules, but replaces exact matching with weighted optimization. The four hard constraints are converted into weak constraints:

```
:~ directed(X,Y,U),
   no_hdirected(X,Y,W),
   u(U).
   [W@0,X,Y,1]

:~ bidirected(X,Y,U),
   no_hbidirected(X,Y,W),
   X < Y,
   u(U).
   [W@1,X,Y,2]

:~ not directed(X,Y,U),
   hdirected(X,Y,W),
   u(U).
   [W@0,X,Y,3]

:~ not bidirected(X,Y,U),
   hbidirected(X,Y,W),
   X < Y,
   u(U).
   [W@1,X,Y,4]
```

An `h*` fact records an edge observed in $\mathcal{H}$, while a `no_h*` fact records an observed absence. The weight W is a non-negative integer derived from the strength of the upstream evidence. The precise priority levels

bioRxiv preprint doi: https://doi.org/10.64898/2026.08.09.743708; this version posted August 14, 2026. The copyright holder for this preprint (which was not certified by peer review) is the author/funder, who has granted bioRxiv a license to display the preprint in perpetuity. It is made available under a CC-BY 4.0 International license.

are configurable; the schematic levels above separate bidirected-structure decisions from directed orientation decisions.

The first two weak constraints penalize edges produced by the candidate that are absent from $\mathcal{H}$. The last two penalize edges present in $\mathcal{H}$ but missing from the candidate's measured-scale image. Unlike an integrity constraint, a weak constraint does not reject the candidate. It records the disagreement as part of its cost.

Strongly supported observed edges receive large omission penalties and are therefore expensive to remove. Weakly supported edges receive smaller penalties and can be removed if doing so yields a more structurally coherent explanation. Conversely, adding an unsupported edge ordinarily receives a high penalty. The resulting objective is a weighted minimum-change problem rather than an exact graph-matching problem.

The trailing numeric field in each cost tuple is a type tag. It distinguishes directed and bidirected mismatch classes associated with the same node pair. Without distinct tags, two ground weak constraints with identical weights, priorities, and node terms can be treated as the same objective element and counted only once.

7.1.9 Evidence weights

Let A denote the lagged association matrix returned by the upstream estimator and let $s_{ij} \in [0,1]$ be the normalized evidence magnitude for the ordered pair (i,j). A representative weighting scheme is

$$w_{ij}^{\text{present}} = \lfloor W_{\max} s_{ij} \rfloor, \qquad w_{ij}^{\text{absent}} = \lfloor W_{\max}(1 - s_{ij}) \rfloor.$$

The first quantity is the cost of omitting an edge that the upstream method selected. The second is the cost of adding an edge that it did not select. A selected edge with strong evidence is therefore expensive to omit, while a rejected edge with very weak evidence is expensive to add.

In the present pipeline, the lagged and contemporaneous evidence matrices are obtained from the PCMCI output [38]. The same ASP formulation can accept scores from other first-order estimators, including Granger-causal or multivariate autoregressive methods. Prior anatomical information can also be incorporated by increasing or decreasing the relevant edge-level penalties.

7.1.10 Density regularization

RnR additionally discourages candidate graphs whose density is incompatible with the expected sparsity of brain networks. A simplified encoding is

```
edge_count(C) :-
    C = #count { X,Y : edge1(X,Y) }.

density_error(E) :-
    edge_count(C),
    target_edges(T),
    E = |C-T|.

:~ density_error(E).
   [E@2,E]
```

The first rule counts the directed edges in the candidate $\mathcal{G}^1$. The second computes the absolute deviation from the target edge count. The weak constraint penalizes that deviation.

This term is a regularizer, not a statement that the true density is known. Its role is to prevent noisy edge evidence from driving the solution toward nearly empty or nearly complete graphs. When used with a hard admissible density window, the implementation can widen the window or fall back to a purely soft penalty if the initial bounds make the instance unsatisfiable.

The cost can therefore be written schematically as

$$C(G) = C_o(G) + C_b(G) + C_d(G),$$

bioRxiv preprint doi: https://doi.org/10.64898/2026.08.09.743708; this version posted August 14, 2026. The copyright holder for this preprint (which was not certified by peer review) is the author/funder, who has granted bioRxiv a license to display the preprint in perpetuity. It is made available under a CC-BY 4.0 International license.

where C_o measures disagreement with the density target, C_b measures bidirected-edge discrepancies, and C_d measures directed-edge discrepancies. Priority levels implement a coarse-to-fine ordering, while the evidence weights resolve trade-offs within each level.

7.1.11 Handling mutual directed pairs

RnR can operate as a meta-solver on the result of an existing causal discovery method. An upstream method without a bidirected-edge primitive may represent dependence due to an undersampling-induced hidden common cause as a mutual directed pair, $i \to j$ and $j \to i$.

For such a pair, RnR can offer $i \leftrightarrow j$ as an additional low-penalty explanation. The solver is not forced to choose the bidirected interpretation. Depending on the remaining structural constraints and evidence weights, it can retain one direction, retain both directions, choose the bidirected edge, or choose a compatible combination.

In the PCMCI front end, contemporaneous links can be represented directly as bidirected edges in the compressed graph supplied to RnR. Both mechanisms serve the same conceptual purpose: they prevent the representational limitations of the upstream estimator from being treated as exact knowledge about the causal-timescale graph.

7.1.12 Solver execution and answer-set decoding

For each participant, the implementation proceeds as follows:

1. PCMCI, using the settings reported in Section 3.1, estimates a lag-one graph and edge-level evidence from the participant's component time series.
2. The PCMCI result is converted to a compressed measured graph $\mathcal{H}$. Directed and bidirected presence and absence facts, their integer weights, the SCC-domain facts, the density prior, and the finite rate domain are added to the ASP program.
3. `clingo` grounds the program and searches jointly over the possible `edge1/2` atoms and the undersampling rate. In exact sRASL mode, an emitted answer set represents a graph–rate pair that satisfies $\mathcal{G}^u = \mathcal{H}$. In RnR mode, the returned models are accompanied by their objective costs.
4. Output is restricted to the predicates carrying the result:

   ```
   #show edge1/2.
   #show u/1.
   ```

   Intermediate atoms used to derive paths, bidirected edges, SCC restrictions, and density are omitted from the displayed answer set.
5. The shown `edge1(X,Y)` atoms are decoded as the directed edges of one causal-timescale graph. The shown `u(U)` atom provides its inferred rate. The Python layer converts these atoms into the internal `gunfolds` graph representation and associates the model with its objective cost.
6. Candidate models are ordered by cost. Let $c_{\min}$ be the lowest cost found for that participant. RnR defines a working candidate pool using the relative criterion

   $$C(G) \leq 1.9c_{\min}.$$

   The $k = 3$ lowest-cost retained members are used in the group analysis, or all retained members if fewer than three are available.

A displayed answer set can therefore take the form

```
edge1(1,1) edge1(1,3) edge1(3,7) edge1(7,2) u(2)
```

which represents a causal-timescale graph containing the four displayed edges and an inferred undersampling rate of two.

bioRxiv preprint doi: https://doi.org/10.64898/2026.08.09.743708; this version posted August 14, 2026. The copyright holder for this preprint (which was not certified by peer review) is the author/funder, who has granted bioRxiv a license to display the preprint in perpetuity. It is made available under a CC-BY 4.0 International license.

7.1.13 Interpretation of the returned set

The outputs of exact sRASL and RnR have related but distinct meanings. In exact sRASL, the returned answer sets form an equivalence class defined by exact equality to $\mathcal{H}$, within the represented rate range and modelling assumptions. Every member is a distinct causal-timescale explanation of the same measured graph.

In RnR, $\mathcal{H}$ is treated as an uncertain estimate. The retained set is therefore a cost-bounded approximation to the exact equivalence class. Every candidate obeys the forward undersampling and structural rules, but candidates may disagree with uncertain features of $\mathcal{H}$ at an explicitly recorded cost. A low cost means that relatively little well-supported information had to be changed; it does not prove that the candidate is the unique causal graph.

For this reason, the downstream analysis does not interpret a single answer set as the uniquely identified neural network. Instead, it retains several low-cost solutions and analyzes their shared and variable features. Edge frequency across solutions measures how consistently an edge is supported within the recovered candidate set. Partitioning the solutions by inferred u additionally makes it possible to determine whether a dominant orientation remains stable across rates or changes when a different temporal relationship between the measured and causal processes is assumed.

This solution-set interpretation is the central connection between the ASP implementation and the empirical analyses in the main text. The solver makes the ambiguity induced by temporal undersampling explicit, while the subsequent edge-frequency and robustness analyses determine which features of that ambiguity are scientifically stable enough to report.

8 Technical Terms

Effective connectivity — the directed, causal influence one brain region exerts over another, as opposed to mere statistical correlation.

Functional connectivity — statistical dependence (typically correlation) between the activity time series of brain regions; undirected.

Temporal undersampling — sampling a process more slowly than its own dynamics, so several fast steps collapse into one measured step.

BOLD signal — the blood-oxygenation-level-dependent fMRI signal, an indirect, slow proxy for underlying neural activity.

Equivalence class — the set of distinct causal graphs that are all equally consistent with the same observed data.

Rate-agnostic structure learning — causal discovery that does not assume the sampling rate matches the causal timescale.

Answer Set Programming — a declarative logic-based method for solving constraint-satisfaction and optimization problems.

Independent component analysis — a data-driven method that separates fMRI data into spatially independent networks (components).

False discovery rate — the expected proportion of false positives among results declared significant; controlled here by Benjamini–Hochberg.

Undersampling-robust / -fragile orientation — an edge direction that stays fixed across inferred sampling rates (robust) versus one that flips (fragile).
